# A Comprehensive Review and Practical Guide of the Applications of Evoked Potentials in Neuroprognostication After Cardiac Arrest

**DOI:** 10.7759/cureus.57014

**Published:** 2024-03-27

**Authors:** Eduard Portell Penadés, Vincent Alvarez

**Affiliations:** 1 Neurology, Hôpital de Sion, Sion, CHE

**Keywords:** mismatch negativity, event-related potentials, middle latency auditory evoked potentials, brainstem auditory evoked potentials, somatosensory evoked potentials, technical aspects

## Abstract

Cardiorespiratory arrest is a very common cause of morbidity and mortality nowadays, and many therapeutic strategies, such as induced coma or targeted temperature management, are used to reduce patient sequelae. However, these procedures can alter a patient's neurological status, making it difficult to obtain useful clinical information for the reliable estimation of neurological prognosis. Therefore, complementary investigations are conducted in the early stages after a cardiac arrest to clarify functional prognosis in comatose cardiac arrest survivors in the first few hours or days.

Current practice relies on a multimodal approach, which shows its greatest potential in predicting poor functional prognosis, whereas the data and tools to identify patients with good functional prognosis remain relatively limited in comparison. Therefore, there is considerable interest in investigating alternative biological parameters and advanced imaging technique studies. Among these, somatosensory evoked potentials (SSEPs) remain one of the simplest and most reliable tools.

In this article, we discuss the technical principles, advantages, limitations, and prognostic implications of SSEPs in detail. We will also review other types of evoked potentials that can provide useful information but are less commonly used in clinical practice (e.g., visual evoked potentials; short-, medium-, and long-latency auditory evoked potentials; and event-related evoked potentials, such as mismatch negativity or P300).

## Introduction and background

Approximately 140.7 people per 100,000 suffer from cardiac arrest each year. Out-of-hospital cardiac arrest affects approximately 356,000 people per year in the United States alone and approximately 275,000 people per year in Europe. Despite early resuscitation efforts, only about 9% of patients survive to reach the hospital [1-4]. Between 80% and 94.4% of these are in a coma on admission to the intensive care unit (ICU) [5-8].

When cardiac arrest occurs, the brain suffers more from anoxia than other vital organs. As a result, up to 70% of patients admitted to the hospital die of brain damage [5,7,9-11]. Despite multidisciplinary efforts to improve survival prospects and enhance the quality of life of survivors, approximately one out of five patients who survive hospital discharge (average 19%, range 3-47%) will have moderate to severe neurological sequelae that will make it impossible to return to work or the premorbid state in general [12].

Estimating neurological prognosis as reliably as possible is thus very important, as it may likely lead to different management approaches. Often, predicting a poor neurological prognosis can result in the withdrawal of appropriate treatment, which can have dire consequences. Conversely, predicting a favorable neurological prognosis generally prompts a more proactive approach. Nevertheless, estimating mortality and determining the appropriate management for these patients are difficult to predict, as the strategies implemented in the ICU in different countries vary considerably according to different cultures and policies [13].

In centers where life-sustaining treatment is not withdrawn, the reported mortality rate is 34.5%, and the reported percentage of patients in a state of unresponsive wakefulness is 29.5% [14]. In centers where life-sustaining treatment is withdrawn in cases of poor neurological prognosis, up to 60% of patients hospitalized in the ICU after cardiac arrest die after life-sustaining treatment withdrawal [9,12-16].

Therefore, the ability to predict the prognosis of these patients during the first few days after cardiac arrest becomes extremely important for planning adequate therapeutic strategies, guiding relatives based on precise information regarding vital and functional prognosis, and also to assess the possible requirement of organ donation [17-23].

Current practice relies on a multimodal approach, which shows the greatest potential in predicting a poor functional prognosis [23-25]. Among the predictive tools, electroencephalography (EEG); biomarkers such as neuron-specific enolase (NSE) and somatosensory evoked potentials (SSEPs); and brain imaging, mainly brain magnetic resonance imaging (MRI) and computed tomography (CT), are particularly important. No single test alone can reliably predict either a good or a poor prognosis [26,27]. However, the combined application of all these tests is reported to increase the specificity of predicting a good neurological prognosis to more than 80% and sensitivity to more than 40% in most studies [28].

Despite various developments related to brain imaging and biological markers, evoked potentials remain among the most widely used and reliable tools; moreover, they only require a non-invasive, bedside procedure. In this paper, we review currently available data and important technical aspects regarding the assessment of neurological prognosis after cardiac arrest using SSEPs. Information provided by other forms of evoked potentials, such as visual evoked potentials; short-, medium-, and long-latency auditory evoked potentials; and event-related evoked potentials, will also be assessed.

## Review

Evoked potentials

Evoked potentials are brain responses that are time-locked to a particular stimulus and reflect neuronal activity from a neuronal assembly [29].

In most evoked potential techniques mentioned in this review, brain activity/response is assessed over the skull in the same way as in EEG. However, EEG is a "passive" recording of brain activity, whereas evoked potentials reflect the brain activity during specific periods of time after an external stimulus is applied. The same stimulus is repeated for a certain amount of time, and the responses are averaged in order to reduce artifacts and brain activity unrelated to the stimulus.

Evoked potentials are generally named based on the applied stimulus, for example, auditory evoked potentials in response to a sonorous stimulus, visual evoked potentials (VEPs) to an optical stimulus, and SSEPs when evaluating the sensory pathway. Depending on the time of recording after each stimulus and the time after which the responses appear, they are referred to as short-, medium-, or long-latency evoked potentials.

Finally, recorded waves are named according to their polarity: "P" for positive responses, usually represented downward on a vertical axis, and "N" for negative responses, usually represented upward on a vertical axis. After a letter is assigned according to polarity, a number is usually added according to the latency at which it normally appears (in milliseconds). Notably, the same wave may have a different name depending on the literature. A good knowledge of evoked potentials is essential to interpret them correctly [30-35].

SSEPs

Anatomical Review

The somatosensory system is mainly composed of two systems: the spinothalamic system and the dorsal column-lemniscal system.

The spinothalamic system is responsible for the transmission of thermal sensitivity, nociception, crude touch, and pressure. Its receptors are mostly located in the skin as free nerve endings of unmyelinated (C) and thinly myelinated (Aδ) nerve fibers and have conduction velocities of less than 2 m/s (C fibers) and approximately 14 m/s (Aδ fibers).

The dorsal column-lemniscal system is responsible for proprioception and sensing position, vibration, pressure, discrimination, and touch. Its receptors are also located mostly at the cutaneous level as free nerve endings of thickly myelinated fibers (Aα and Aβ) and have conduction velocities of 30-80 m/s.

It is important to note that only the dorsal column-lemniscal system function is evaluated using SSEPs [35-42].

Stimulation and Recording

**Figure 1:**
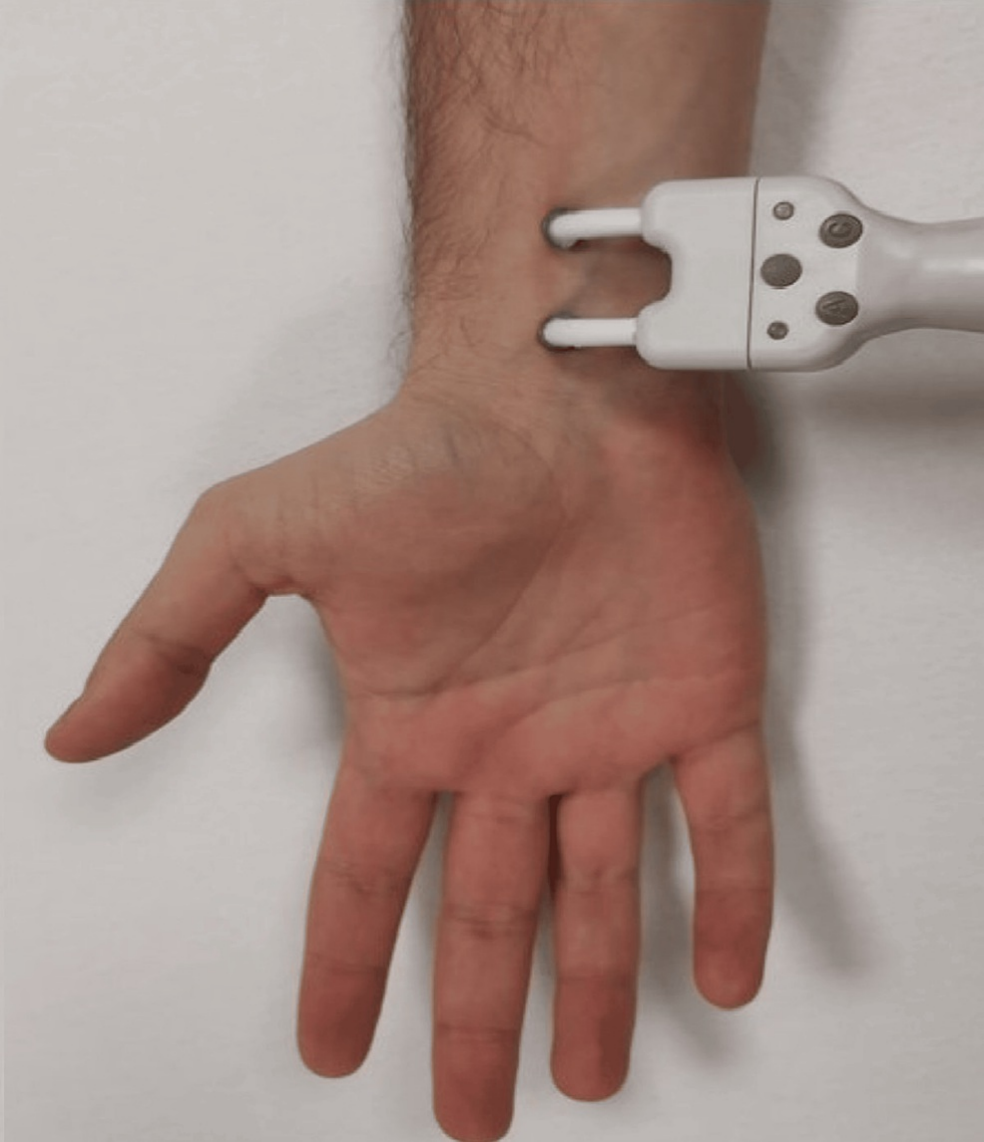
Stimulation of the median nerve at the wrist level The anode (positive electrode) should be at the distal position. Alternatively, subdermal needle electrodes can be used. Movement of the thumb should be confirmed to ensure sufficient intensity of stimulation (while being careful with curare).

It is recommended to stimulate the median nerve (Figure 1) at the wrist level, if possible, using a bipolar electrical stimulator with the anode placed distally and the cathode placed proximally to avoid a questionable anodal block. In case access at the wrist level is not feasible (e.g., arterial line in the ICU setting), the nerve could be stimulated proximally in the forearm or at the brachial plexus level; however, this would modify the latency of the results, which should be analyzed carefully in such cases [43].

The stimulus is a square wave current with a short pulse width (0.1-0.5 ms). To obtain a better definition and amplitude of SSEP waves useful in post-anoxic coma patients, a stimulus frequency lower than 5 Hz, ideally between 2 and 3 Hz, should be used [44,45].

For correct interpretation, it is essential not to understimulate the peripheral nerves. Therefore, it is necessary to stimulate the sensitive nerve between two and four times its sensory threshold or above the motor threshold in the case of mixed nerves. For the median nerve, a distal reproducible muscle twitch should be observed. If muscle relaxants have been used, the muscle twitch will be suppressed; in such cases, an intensity of approximately 20 mA is recommended. The intensity used should be the same on both sides.

For comatose patients in the ICU, it is advisable to use subdermal needle electrodes for recording to minimize impedance and reduce background noise. Ideally, impedances should be lower than 3-5 kΩ [34,46].

A total recording time window of 50 ms is recommended. The bandpass should be set with a high-pass filter of less than 3 Hz and a low-pass filter of over 2000-3000 Hz.

To demonstrate the reproducibility of the responses, it is recommended to average at least two blocks of 500 stimuli for each side, although some authors have proposed calculating the average of three sets of more than 200 responses [30,42,47-51].

 

 

 

 

Timing and Other Considerations

After cardiac arrest, a significant improvement in evoked potentials is generally noted during the first 24 hours after the return of spontaneous circulation (ROSC). Therefore, it is generally recommended that SSEPs be recorded at least 24 hours after cardiac arrest, although some groups have proposed waiting 48 hours before SSEP recording [52-56]. It is important to note that SSEPs are considerably resistant to hypothermia and to most sedative drugs, muscle relaxants, and anesthetics frequently administered in the ICU. SSEPs continue to be present down to 22°C (severe hypothermia), which is typically not reached in the ICU. Between 32°C and 36°C, SSEPs remain a reliable sign [30,57-62].

In addition, in patients without brain injury, SSEPs remain present even with sedation sufficient for isoelectric EEG [42]. This level of sedation is rarely reached in the management of post-cardiac arrest patients.

This reliability of SSEPs, regardless of body temperature or sedation, is very convenient in the ICU setting.

Recording Electrodes, Main Waves, and Their Generators

**Figure 2:**
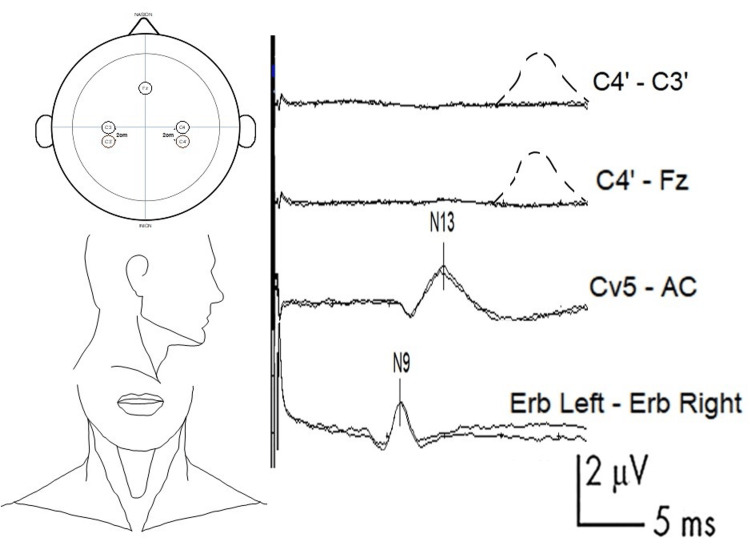
Example of SSEP with absent N20 response and recommended electrode placement and normal curves First channel (bottom line): N9 (normal value: approximately 9 ms) is generated in the trunks of the brachial plexus. This wave is recorded in the ipsilateral Erb-contralateral Erb channel. The Erb point is located within the angle formed by the posterior border of the clavicular head of the sternocleidomastoid muscle and the clavicle, 2-3 cm posterior to the clavicle. Second channel (middle line): N13 (normal value: approximately 13 ms) is generated by the postsynaptic activity of the dorsal horn of the cervical spinal cord. This wave is recorded from Cv5 (placed on the skin over the fifth cervical spine), and the AC (placed in the anterior cervical region) is used as the reference. Top channel: example of an absent N20. The dashed line represents the expected curve, which was generated in the primary sensitive cortex of the postcentral gyrus (normal value: approximately 20 ms). This wave is recorded from C3' and C4' (2-3 cm behind C3 and C4) (contralateral side as the active electrode, ipsilateral side as the reference) or by using Fz as the reference instead. SSEP: somatosensory evoked potential

For recording electrode placement and normal wave patterns, see Figure 2. The nomenclature of skull electrodes is based on the international EEG 10-20 system [63,64].

The N9 wave (negative polarity, normal value: approximately 9 ms) is found to be generated in the trunks of the brachial plexus. This wave is recorded in the ipsilateral Erb-contralateral Erb channel. It can also be obtained by recording from the ipsilateral Erb-Fz. The Erb point is located within the angle formed by the posterior border of the clavicular head of the sternocleidomastoid muscle and the clavicle, 2-3 cm posterior to the clavicle.

Following the direction of the afferent pathway, the next wave to focus on is the N13, which corresponds to the segmental postsynaptic activity of the dorsal horn of the cervical spinal cord. This wave is best measured using an electrode placed on the skin over the fifth cervical spine (Cv5), although some researchers recommend using the sixth cervical spine (Cv6). An electrode placed in the anterior cervical (AC) region is used as a reference; alternatively, the contralateral Erb or Fz can be used as references. Although other SSEP waves can provide useful information for different pathologies, they are not essential for the management of post-cardiac arrest neurological prognosis.

The wave that provides more information about the prognosis of post-anoxic patients in a coma is N20. This wave is generated in the primary sensitive cortex in the postcentral gyrus. To record it, electrodes must be placed 2-3 cm behind C3 and C4 (or halfway between C3 and P3 on the left and C4 and P4 on the right), called C3' and C4'. The contralateral side will be used as the active electrode (CPc) and the ipsilateral side (CPi) as the reference or, alternatively, with the active electrode in CPc and with the reference in Fz or in the contralateral Erb. It is important to know that if we use any of these last two references, when dealing with far-field potentials, a subcortical wave called N18 can also be observed, whose generator is thought to reflect the delayed activity within the thalamus or the caudal part of the thalamocortical projection fibers. This wave is not observed from C3' vs C4', as the latter are near-field.

Finally, as an interesting element to highlight regarding short-latency SSEPs for the prognosis of post-anoxic patients, the P25 component, obtained from the same channels, has been identified as the major positive peak following N20 [30,34,44,51,65,66].

Prognostic Value

The bilateral absence of N20 is considered the most reliable marker of poor outcome, with a relatively low sensitivity (30.5%) for predicting a poor outcome, but a specificity of 100% and a false-positive rate of 0.007 (confidence interval (CI), 0.001-0.047).

It has been reported that assessing the absence of the N20-P25 component, instead of the absence of the N20 wave alone, increases the sensitivity for predicting a poor outcome to 71.6% while maintaining a specificity of 100% [28,67-70].

The power of the absence of the bilateral N20 response to predict poor prognosis has been well demonstrated. However, the ability of the presence of N20 waves or their amplitudes to predict a good outcome had not been extensively investigated until recently. Some studies have shown that the presence of a bilateral N20 response is predictive of a good outcome (Glasgow Outcome Scale (GOS) score 4 or 5) or awakening in 86-93% of patients, but there is insufficient evidence in the literature [30,71,72].

Initially, the N20 wave had been analyzed as a dichotomous variant (present/absent). Attention has now also been focused on the prognostic implications of the amplitude of this wave as a continuous variable. However, given that the stimuli and recording techniques differed among various studies, the proposed thresholds are highly variable, and standardization is required.

Very wide ranges of low voltage amplitude thresholds (between 0.4 and 1.0 μV) have been described, increasing the sensitivity of predicting a poor outcome to around 50% while maintaining a specificity of up to 100%. In a recent retrospective multicenter study, a threshold of 0.5 μV was utilized in place of the criterion of bilateral cortical SSEP absence. This modification yielded an elevated sensitivity for the prediction of an unfavorable outcome, increasing from 30.3% to 42.9% [73-76].

It should be noted that studies differ in the way the amplitude is calculated, with the difference between baseline and N20 (peak-to-baseline) or between N20 and P25 (peak-to-peak) being considered for measurement. A recent study pointed out that the N20-P25 amplitude had a higher prognostic value compared to the N20-baseline amplitude [77,78].

With the same limitations as for the lower threshold, it has been reported that large amplitudes are related to a better prognosis. Amplitudes >2.3-4 μV predict a favorable outcome with high specificity (85-96%) but moderate sensitivity (30-61%) [29]. In another study, the combination of N20 wave amplitude ≥3 μV and the presence of a continuous and normovolted EEG was reported to increase the sensitivity for good outcome prediction [79]. Nevertheless, prospective external validation of the lower and higher thresholds remains necessary.

The observation of an asymmetric N20 response has rarely been reported in the literature. It should be evaluated with caution, and it is worth considering the possibility of a technical failure (due to either an error in the preparatory process or artifact noise) or a focal nerve or brain dysfunction.

It should be noted that for the correct interpretation of the absence of the N20 wave, the presence of N9 and N13 responses is mandatory. If N9 and N13 responses are not present, the absence of N20 might not be due to a cortical impairment and is thus not interpretable.

There is a limitation criticized in most studies regarding the theory of the self-fulfilling prophecy. Specifically, in numerous studies examining the predictive value of poor prognosis of SSEPs, they have been utilized as a criterion for terminating life-sustaining treatment. This approach can result in bias and lead to erroneous conclusions. Although important multicenter studies conducted without the withdrawal of life-sustaining treatments have shown concordant results, isolated cases of absent N20 responses with good functional outcomes have also been described, although these have generally been highly debated [28,67,69,80-83].

Auditory evoked potentials

Auditory evoked potentials are the neurophysiological tool used to evaluate the auditory pathway and can be measured by recording the electrical changes in response to sonorous stimuli.

Depending on the recording time, various specific parts of the auditory system can be evaluated. Immediate responses after auditory stimulation (10 ms) are used to analyze the auditory pathway from the cochlear nerve to its passage through the brainstem. These are known as brainstem auditory evoked potentials (BAEPs) and are the most commonly used in daily clinical practice.

Middle-latency auditory evoked potentials (MLAEPs) are recorded during the first 100 ms and are used to evaluate the auditory pathway projecting from the medial geniculate body to the primary auditory cortex.

Long-latency or late auditory evoked potentials are used to evaluate more complex neural networks and are especially useful for reflecting cognitive attention tasks [51].

BAEPs

Stimulation and recording: BAEPs are far-field responses evaluated in the first 10 ms after a monaural auditory stimulus generated using headphones. The stimulus intensity should be set at 80-90 decibel hearing level (dBHL) on the stimulated side, with bone-conduction masking noise in the contralateral ear at an intensity of approximately 20-30 dBHL lower than in the ear being stimulated.

Depending on whether the diaphragm of the earphone moves in the direction of the patient's ear owing to a pressure increase or in the opposite direction, stimulus polarity is defined as positive or negative, respectively. Change in polarity can modify the latencies; therefore, using a single polarity for the same patient is generally recommended, although, as we will see below, the latencies have little implication for the prognosis of post-anoxic patients.

**Figure 3:**
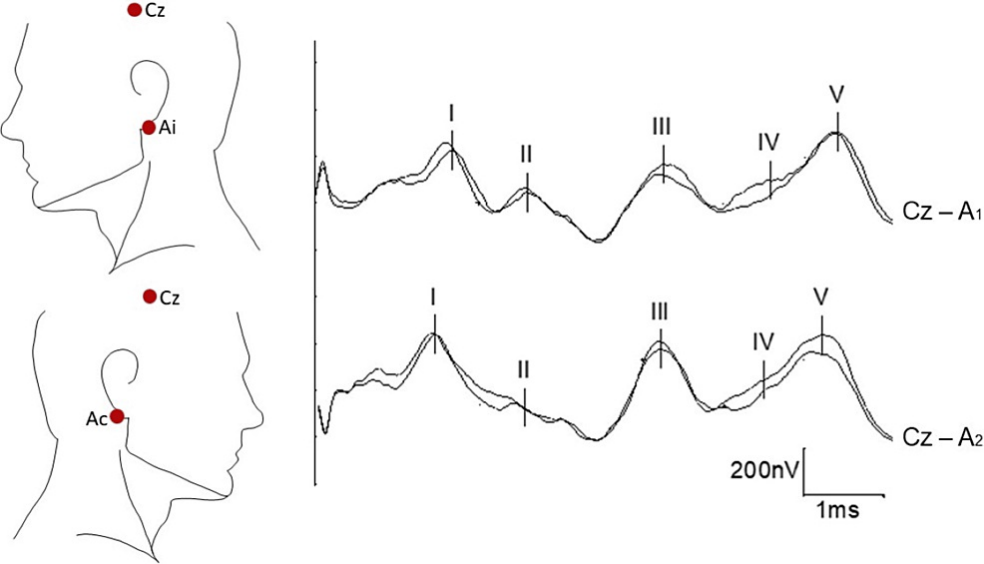
BAEPs: recommended electrode placement and normal curves The reference for both channels will be Cz (the skull), with the active electrode on the ipsilateral earlobe (Ai) in one channel and on the contralateral earlobe (Ac) in the other channel. The mastoid can be used instead of the ear (called in this case Mi: ipsilateral or Mc: contralateral). The five main waves are named sequentially using Roman numerals in the order of appearance from peripheral to central (I, II, III, IV, and V). BAEPs: brainstem auditory evoked potentials

For recording in the ICU, we preferably use two channels and subdermal needle electrodes (Figure 3). The reference for both channels will be Cz (the skull), with the active electrode on the ipsilateral earlobe (Ai) in one channel and on the contralateral earlobe (Ac) in the other channel. Sometimes, the mastoid is used instead of the ear and is referred to as M (Mi: ipsilateral or Mc: contralateral). As opposed to SSEPs and EEG, for BAEPs, we define positivity as upwards and negativity as downwards.

The bandpass should be set between 100 Hz and 1500 Hz, and two averages per side with 1200-2000 stimuli per trial should also be performed to demonstrate the reproducibility of the results. The stimulus frequency can vary between 8 Hz and 24 Hz. Electrical contact impedance should be kept below 5 kΩ [33,49,84-86].

BAEPs are only slightly affected by sedatives and can be recorded with moderate doses. To eliminate muscle-related artifacts, transient neuromuscular blockades can be administered [30].

Although there are no specific guidelines on when to record BAEPs, they are typically performed after the first 24 hours following a coma. This enables us to utilize the assistance available in the ICU for applying both SSEPs and BAEPs, which helps to optimize time.

Main waves and their generators: With regard to BAEPs, we find five main waves that are named sequentially using Roman numerals in order of appearance from peripheral to central (I, II, III, IV, and V) (Figure 3), instead of according to their polarity and latency, as with SSEPs.

Wave I reflects the nerve action potential of the auditory portion of the eighth cranial nerve, also called the cochlear nerve, probably from its proximal portion. Wave II may be generated in or near the cochlear nucleus in the medulla oblongata. Wave III is generated in the ipsilateral superior olivary complex and the trapezoidal body located in the lower pons. Wave IV has multiple bilateral generators in the rostral part of the pons and the caudal part of the mesencephalon. Wave V is generated in the contralateral distal lateral lemniscus or in the inferior colliculi in the caudal mesencephalon.

Occasionally, we may find waves VI and VII, but these are much more inconsistent and are not used in clinical practice. Their presence or absence has no known clinical significance [85-88].

Prognostic value: BAEPs have rarely been studied as prognostic factors after cardiac arrest except in cases of brain death.

The absence of the central BAEP waves (III-V) is related to an unfavorable outcome with high specificity but low sensitivity. Furthermore, preserved BAEPs are not predictive of a good prognosis [28,51,87-89]. The brainstem is more resistant to hypoxia than the cerebral cortex. Therefore, the preservation of brainstem function cannot predict the preservation of cortical function, which is also crucial for a good neurological prognosis. However, the absence of BAEPs is associated with severe brain damage.

Other parameters, such as response latency, cannot differentiate survivors from non-survivors.

The main application of BAEPs for the prognosis of post-anoxic patients thus lies in the assessment of brainstem integrity for the diagnosis of brain death and, most importantly, in confirming the integrity of the auditory system at the peripheral and brainstem levels before assessing MLAEPs and late auditory evoked potentials [28,71,87-89].

MLAEPs

Stimulation and recording: MLAEPs are near-field responses that appear within the first 100 ms of a monaural auditory stimulus. Their waves originate in the auditory pathway between the projections of the medial geniculate body to the primary auditory cortex.

The stimulus characteristics of MLAEPs are similar to the parameters defined for BAEPs, except for stimulus frequency. To favor good wave definition, it is necessary to stimulate at a frequency lower than 10 Hz; the recording time will be up to 100 ms instead of 10 ms.

**Figure 4:**
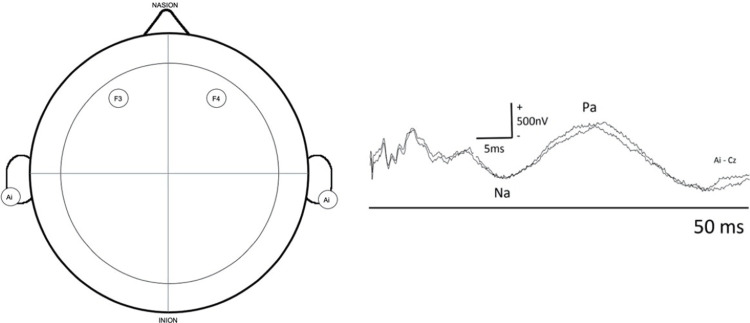
MLAEPs: recommended electrode placement and normal curves Two channels with the active electrode located over both primary auditory cortices (F3 and F4) and with a common reference to the ipsilateral earlobe or mastoid. A negative wave called Na, of debated origin but probably thalamic, appears at approximately 17 ms after the stimulus, and a second positive wave at around 30 ms after the stimulus, called Pa, is generated in the primary auditory cortex. MLAEPs: middle-latency auditory evoked potentials Courtesy of Dr. Misericordia Veciana and Dr. Jordi Pedro Pérez, Neurophysiology Department, Hospital Universitari de Bellvitge, Barcelona, Spain

For recording, it is recommended to use two channels with the active electrode located over both primary auditory cortices (F3 and F4) and with the common reference on the ipsilateral earlobe or mastoid (Figure 4). However, some researchers have used Cz or Fz as the active electrodes instead of F3/F4.

MLAEPs can be recorded simultaneously with BAEPs as long as the stimulus frequency is maintained appropriately. A four-channel montage using different recording times for the two channels of BAEPs (10 ms) and MLAEPs (100 ms) is necessary [72,89-95].

However, it should be noted that MLAEPs are poorly resistant to benzodiazepines and other sedative drugs and anesthetics, thereby being substantially affected by the administration of these compounds [96-101].

Main waves and their generators: For MLAEPs, two waves are mainly analyzed. A negative wave called Na, which is of debated origin but is probably thalamic, appears approximately 17 ms after the stimulus, and a second positive wave, called Pa, is generated in the primary auditory cortex and appears around 30 ms after the stimulus.

It should be noted that a post-auricular muscle artifact due to an audiomyogenic reflex response can often appear with a latency of approximately 10-14 ms and can hinder or alter MLAEP interpretation. If this response interferes with the test, curare can be used to abolish it [72,89-95].

Prognostic value: In post-anoxic patients, the absence of a bilateral Na/Pa response is associated with a poor outcome with practically 100% specificity but with low sensitivity (37%). However, the presence of MLAEPs does not necessarily predict a good outcome [72,90].

The combination of MLAEPs and SSEPs could improve the assessment of neurological prognosis in post-anoxic comatose patients [30,102]. Since there is insufficient evidence in the literature on this subject, isolated interpretation of MLAEPs without SSEPs is not recommended. It also needs to be recalled that preserved BAEPs are necessary to assess MLAEPs.

There are no specific recommendations regarding the timing of MLAEPs. They are usually recorded at the same time as SSEPs and BAEPs, after the first 24-48 hours following coma onset [31,72,89-95].

Long-Latency Event-Related Potentials (ERPs)

Mainly from the primary auditory cortex, the auditory pathway diverges into a complex neural network. Therefore, long-latency ERPs are considered to reflect a cognitive attention task. This could be seen as an approach for improving cognitive function in unresponsive patients.

The presence of ERPs is generally related to cognitive evaluation, because a certain attention capacity of the patient is necessary for their presence. Therefore, ERPs are highly sensitive to drugs that may alter a patient's level of consciousness. Since neuromuscular blockers do not affect the level of consciousness, their use does not seem to alter these responses and could be useful in improving the quality of the recording and eliminating muscle responses that could confound the interpretation.

Several different auditory stimuli have been proposed, although very few are used in clinical practice for coma prognosis. Those that have gained relatively more importance are the so-called mismatch negativity (MMN) and the P3 response evoked by the subject's own name (P3 to SON). However, literature on these evoked potentials remains rather limited [103].

MMN

To understand MMN, it is necessary to define the concept of the oddball paradigm. This involves the application of frequent standard identical sound stimuli and alternating them randomly, and occasionally or rarely, with sound stimuli that differ in intensity, frequency, or duration [104].

For auditory MMN, the long-latency response (>100 ms) obtained after the standard repetitive responses is compared to the responses obtained after deviant tones. As the patient does not actively participate in this paradigm, i.e., it is a passive paradigm, MMN is applicable in comatose patients, although it is not assessable in the case of sedative medication, as mentioned above.

MMN is thus a pre-attentive and automatic response that reflects the capacity of the patient's brain to detect relevant stimuli over background noise, which implies a certain capacity for reaction and short-term memory for these changes to be detected [31,105-107].

Stimulation and recording: For sound stimulation, it is necessary to calibrate two different sound stimuli.

Both stimuli are pure tones of 800 Hz and tone-burst of 80 dBHL and have short rise and fall times (<10 ms). The standard sound stimuli have a duration of 75 ms. The deviant sound has a duration of 35 ms. There is a difference in frequency of 500 Hz for the standard and 1000 Hz for the deviant tones.

In general, between five and eight series of stimuli are necessary to reproduce the differences, with approximately 200 stimuli in each series, of which approximately 172 are standard (86%) and 28 are deviant (14%), presented in a random order.

An excessive increase in the number of stimuli may create habituation and decrease MMN amplitude. However, in some studies, larger (up to 22 blocks) but shorter (100 stimuli in each series) series were used.

Hypodermic needle electrodes are placed according to the international 10-20 system, with the active electrode in the midline (Fz, Cz, or Pz; preferably Fz) and with linked or unlinked reference electrodes in the mastoids or earlobes.

A period of up to 600 ms is recorded, of which 100 ms is pre-stimulus and 500 ms is post-stimulus. The band-pass frequency limit range is set between 0.1 Hz and 30 Hz [31,105-109].

**Figure 5:**
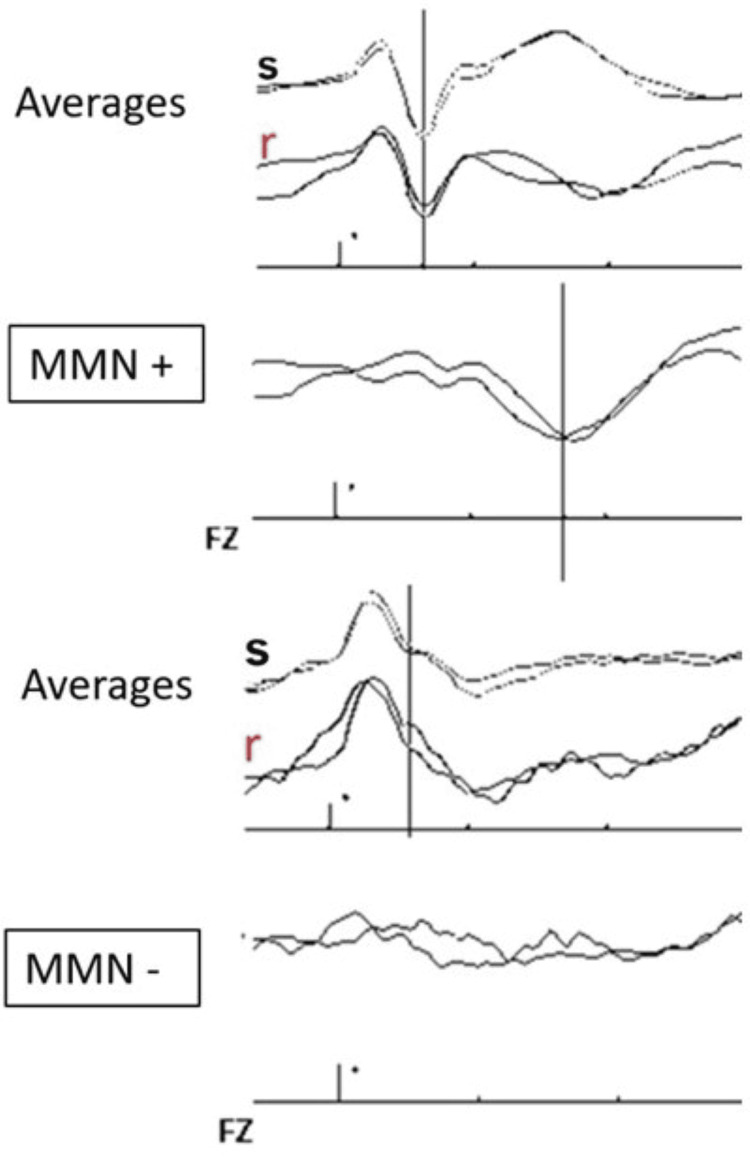
MMN curve Figure 5 shows two examples: on top, a patient with an MMN present, and on the bottom, a patient with MMN absent. We observe the averages after a series of standard stimuli (s) and the responses after rare stimuli (r). Subtracting the standard from the rare averages yielded the MMN. The first large negative wave observed at approximately 100 ms is called N100 or N1 and is generated in the auditory cortex, the presence of which is required to obtain MMN. The electrodes are placed according to the international 10-20 system, with the active electrode in the midline (Fz, Cz, or Pz), preferably in Fz, and with reference in the mastoids or earlobes, linked or not linked. MMN: mismatch negativity Courtesy of Dr. Misericordia Veciana and Dr. Jordi Pedro Pérez, Neurophysiology Department, Hospital Universitari de Bellvitge, Barcelona, Spain

Waves and interpretation: Following a sound stimulus in a preserved auditory pathway, a large negative wave at approximately 100 ms, generated in the auditory cortex and called N100 or N1, is observed (Figure 5). The presence of this wave is essential for evaluating MMN.

The responses obtained after the standard stimuli are averaged and compared with the average of the long-latency responses obtained after the deviant stimuli. The first standard stimulus after each deviant stimulus is not averaged so as not to alter the results.

The MMN is thus this difference in long-latency auditory responses. However, there is one relevant technical issue. When comparing the responses obtained after the standard and deviant stimuli, we are comparing two groups of averages with very different numbers of records. Therefore, the response to deviant stimuli may be more biased by ambient noise artifacts in this group.

In order to try to minimize this bias, one study proposed a variation of the analysis technique using an automated discriminatory paradigm. This study only recorded the response to the deviant stimulus by subtracting the response to the previous standard stimulus, thus obtaining a set of difference waves using an automated single-trial procedure for MMN analysis. This has only been tested in a clinical study in the ICU environment, which showed that this automated discriminatory paradigm significantly improved early coma prognostic accuracy after cardiac arrest. However, further studies are needed before it can be implemented as a routine technique [110,111].

MMN responses involve at least two main intracranial processes: one at the supratemporal level, presumably related to the ability to perceive differences between stimuli, and the second, predominantly in the frontal areas, related to the involuntary attention generated by stimulus changes.

Prognostic value: The absence of an N100 response, regardless of the presence of MMN, is considered predictive of an unfavorable outcome.

The presence of MMN predicts awakening or return to consciousness with a specificity of more than 90% and a positive predictive value of 100% in post-anoxic patients. However, the sensitivity remains low, although it varies greatly between studies (27-100%).

One limitation also lies in the meaning of consciousness, as it encompasses the entire state of consciousness, except for death or the permanent vegetative state. This technique strongly predicts a certain degree of attention/awakening, although this may be minimal and does not necessarily indicate a good functional outcome. Approximately 35% of patients in the minimally conscious state have MMN [71,112-114].

P3 Responses Evoked by Subject's Own Name (=SON)

In this ERP, a new stimulus concept is added, called novel stimuli or Novels (which is why P3 to SON is also known as novelty P3 or nP3). This technique involves adding a third sound stimulus to the two stimuli already used for MMN. This new stimulus appears even less frequently and consists of an unexpected and noticeable stimulus that generates complex attention-orienting processes. This may reflect a higher level of cortical activation than that in MMN.

The novel stimulus can be any extremely deviant tone, such as music, bell ringing, or dog barking, as well as vocal stimuli. SON has gained weight as a novel stimulus because it activates a large number of neurons and also has an emotional component. Generating the sound of the SON in a familiar voice has also been proposed to increase the emotional aspect of the stimulus.

Like MMN, P3 to SON does not require any activity by the patient (passive paradigm); therefore, it can also be performed on comatose patients and is altered by sedative medication.

Stimulation and recording: For the recording of P3 to SON, the same parameters are used as for MMN, with the addition of a new auditory stimulus, which will be presented randomly (on average, one out of 20 times, 5%).

To minimize background noise artifacts, at least 50 SON stimuli are required. The parameters of the standard and deviant sounds are maintained.

Between five and eight stimulus series of approximately 170 stimuli each (132 standard, 28 deviant, 10 SON) should be performed.

The electrodes are placed in the same way as for MMN, but with an analysis time lengthened to 1100 ms (100 ms pre-stimulus, 1000 ms post-stimulus). Several oddball paradigms have been developed to simultaneously record MMN and P3 to SON.

Waves and interpretation: In response to these different alternating stimuli, we obtain a complex positive wave with a peak at approximately 300-350 ms (P3 or P300), which has two distinct components with different topographies.

Between 220 ms and 300 ms, we find the first component, maximum in the central regions, called early P3 or P3a, which is associated with alerting.

The second stage of the wave, between 300 and 380 ms, is called the late P3 or P3b. It is maximal in the parietal cortex and is related to stimulus categorization, significance as a target, and degree of information delivery.

In one study, late parietal positivity was sometimes detected in the 450-850 ms window, showing a larger amplitude when the SON was uttered by a familiar voice. This may be associated with recollection processes [115].

Prognostic value: Few studies have evaluated the prognostic value of P3 to SON in post-anoxic comatose patients. A study by Fisher et al. in 2008, in which P3 to SON was assessed in 50 comatose patients (20 due to anoxia), showed a similar specificity to MMN for predicting awakening (85%), but higher sensitivity (71% for nP3, 42% for MMN), a positive predictive value of 81%, and a negative predictive value of 76%.

Again, the P300 is a remarkable marker for awakening prediction, but like MMN, the concept of awakening is very broad, and the presence of this wave does not exclude mild to severe neurological disability [30,113-118].

VEPs

VEPs are the neurophysiological tool used to evaluate the visual system and assess visual integrity from the retina to the primary visual cortex. The integrity of multiple ocular parameters can be evaluated depending on the available apparatus and technique used.

Different types of visual stimuli have been described, such as pattern-reversal VEPs elicited by checkerboard stimuli and pattern onset/offset VEPs elicited by checkerboard stimuli. With these techniques, better-quality VEPs are obtained, but patient collaboration and attention are required; therefore, they are not applicable in the case of comatose patients.

Another way of eliciting evoked potentials is by using light-emitting diode (LED) goggles. The responses to this type of stimulus are not as constant as those to checkboard stimuli, but they do not require patient collaboration and can be performed in post-anoxic patients in a coma even when their eyes are closed.

The advantage of VEPs lies in the fact that they are associated with a supramedullary neurological pathway; thus, alterations in the peripheral nervous system, medulla, or brainstem do not alter the results.

Stimulation and Recording

A binocular flash is applied from LED goggles placed on both eyes of the patient as the visual stimulus. The luminance is approximately 30 cd/m2 and the frequency is 1 Hz or 1.3 Hz. Both eyes can be stimulated simultaneously or individually [30]. The impedance should be less than 5 kΩ, ideally <3 kΩ. A bandpass of 0.5-100 Hz should be employed. Hypodermic needle electrodes should be used for recording.

The recording electrodes are placed following the international 10-20 system at the Oz location, with the reference at Fz.

Alternatively, some researchers have proposed the use of a four-channel setup, with one channel to record from the active electrode in the outer canthus of the stimulated eye, two channels with O1 and O2 to record the cortical response, and the last Cz channel, with a common reference in all four channels against linked-ear instead of Fz, as this may decrease electroretinographic contamination.

To better define the responses and reduce artifacts, 60-100 sweeps should be performed at least twice to ensure reproducibility. The cortical response is analyzed 200-500 ms after the stimulus [119-121].

Waves and Interpretation

In the flash VEP, we obtain a wave-shaped response with three negative waves intercalated with three positive waves (N1-P1-N2-P2-N3-P3), which are labeled in increasing order using Roman numerals (I, II, III, IV, V, and VI, respectively). These waves appear in the first 250 ms after the flash stimulus. The main waves were those with negative components (I, III, and V). Wave I has a retinal origin and appears at approximately 50 ms, while wave III appears at approximately 75-95 ms and has an occipital origin. The latencies between individuals vary widely [119-121].

Prognostic Value

Wave I, which has a retinal origin and is highly resistant, is used, like BAEP wave I, as a control for recording quality in cases of markedly altered VEP. Wave III is the most reliable wave.

As encephalopathy of nonspecific etiology worsens, the latency of wave III is prolonged until it finally disappears.

Few studies have investigated the prognostic value of VEPs in post-anoxic patients. Studies performed in 1998 by Guérit et al. showed that wave III preservation with a normal latency or <120 ms was associated with a good outcome [122]. In 2015, Heinz and Rollnik published a study in which VEPs were recorded in 93 post-anoxic comatose patients. It was observed that a prolongation of wave III latency >125 ms was associated with a poor outcome, although this latency was not related to the degree of consciousness [123].

Another study evaluated, among other things, the value of VEPs in the neurological prognosis of post-anoxic patients during and after target temperature management (TTM). This study concluded that during TTM, the absence of VEPs predicted a poor neurological outcome with a sensitivity of 44% and a specificity of 96%. After rewarming, VEPs predicted a poor neurological outcome with a sensitivity of 47% and a specificity of 100% [57].

When SSEPs and VEPs were combined, the prognostic accuracy increased slightly compared to that of SSEPs alone [57,119-121,123-127].

## Conclusions

Despite the evolving landscape of technological tools in neurology, evoked potentials remain indispensable for cardiac arrest prognostication due to their bedside applicability, non-invasiveness, and resistance to sedation and hypothermia. Mastering the diverse array of available evoked potentials offers valuable alternatives for clinicians.

Sensory-evoked potentials, particularly the N20 response, hold significant prognostic value, but especially when absent. The increasing significance of the amplitude of the N20 response also appears to enhance the prognostic value in terms of a favorable outcome, although much scientific evidence is still lacking, and at least for the time being, it remains a supportive tool.

ERPs provide insights into complex cognitive capacities and might be a marker of good prognosis.

Although other evoked potential techniques may have limited relevance in routine clinical practice, awareness of their potential utility in specific cases could be useful. Standardizing evoked potential techniques emerges as a key future objective for enhancing comparability across studies and achieving result homogenization.
